# Effects of Nt-truncation and coexpression of isolated Nt domains on the membrane trafficking of electroneutral Na^+^/HCO_3_^–^ cotransporters

**DOI:** 10.1038/srep12241

**Published:** 2015-07-20

**Authors:** Deng-Ke Wang, Ying Liu, Evan J. Myers, Yi-Min Guo, Zhang-Dong Xie, De-Zhi Jiang, Jia-Min Li, Jichun Yang, Mugen Liu, Mark D. Parker, Li-Ming Chen

**Affiliations:** 1 Department of Biophysics and Molecular Physiology, Key Laboratory of Molecular Biophysics of Ministry of Education, Huazhong University of Science and Technology School of Life Science and Technology, Wuhan, Hubei 430074, China; 2 Department of Physiology and Biophysics, School of Medicine, University at Buffalo: The State University of New York, Buffalo, NY 14214, USA; 3 Department of Physiology and Pathophysiology, School of Basic Medical Sciences, Peking University Health Science Center, Beijing 100091, China; 4 Department of Genetics and Developmental Biology, Key Laboratory of Molecular Biophysics of Ministry of Education, Huazhong University of Science and Technology School of Life Science and Technology, Wuhan, Hubei 430074, China

## Abstract

The SLC4 genes are all capable of producing multiple variants by alternative splicing or using alternative promoters. The physiological consequences of such diversity are of great interest to investigators. Here, we identified two novel variants of the electroneutral Na^+^/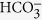 cotransporter NBCn1, one full-length starting with “MIPL” and the other Nt-truncated starting with “MDEL”. Moreover, we identified a new promoter of *Slc4a10* encoding NBCn2 and a novel type of Nt-truncated NBCn2 starting with “MHAN”. When heterologously expressed, the new NBCn1 variants were well localized to the plasma membrane and exhibited characteristic NBCn1 activity. However, MHAN-NBCn2 was poorly localized on the plasma membrane. By deletion mutations, we identified the Nt regions important for the surface localization of NBCn2. Interestingly, coexpressing the full-length NBCn2 greatly enhances the surface abundance of the Nt-truncated NBCn2. Co-immunoprecipitation and bimolecular fluorescence complementation studies showed that the full-length and Nt-truncated NBCn2 interact with each other to form heterodimers in neuro-2A cells. Finally, we showed that the isolated Nt domain interacts with and enhances the surface abundance of the Nt-truncated NBCn2. The present study expands our knowledge of the NBCn1 and NBCn2 transcriptome, and provides insights into how the Nt domain could affect transporter function by regulating its membrane trafficking.

Bicarbonate transporters play critical roles in pH regulation, a house-keeping function of the body. A great diversity of 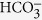 transporters has been recognized during the past decades. The first degree of diversity of 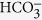 transporters arises from divergence of genes at the genomic level. A series of 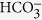 transporter genes have been identified, primarily distributed in the solute carrier 4 (Slc4) and Slc26 families (for review, see refs 1,2). The Slc4 family bicarbonate transporters are broadly distributed in diverse tissues and play a wide variety of physiological roles. Dysfunction of these transporters are associated with a broad spectrum of diseases in human (for review, see ref. 1). The Slc4 family consists of 10 genes, of which nine have been demonstrated to encode 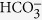 transporters. Among the nine 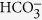 transporter members, three are well-established Na^+^-independent anion exchangers (AE1–3), five are Na^+^-coupled 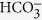 transporters (NCBTs), including NBCe1, NBCe2, NBCn1, NBCn2, and NDCBE. Although originally characterized as an electroneutral Cl^–^/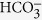 exchanger^3^,^4^, the transport mode (Na^+^ dependence) of Slc4a9 (provisionally designated as “AE4”) remains controversial (for review, see ref. 1).

The second degree of diversity in the Slc4 family of 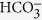 transporters arises from the ability of each gene to express multiple variants via alternative transcription and alternative splicing (for review, see refs 1,5). Among the Slc4 family members, the electroneutral Na^+^/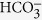 cotransporter NBCn1, encoded by *Slc4a7*, exhibits the highest degree of diversity in its expression products. *Slc4a7* has two alternative promoters plus five known major cassette exons that can be alternatively spliced. The *Slc4a7* gene is known to produce up to 16 major full-length NBCn1 variants, designated as NBCn1-A through -P in the order of discovery^6^,^7^,^8^,^9^. These NBCn1 variants have two types of amino-termini (Nt) starting with “MEAD” versus “MERF” (each representing the first four residues of the Nt end) derived from the usage of either of the two alternative promoters of *Slc4a7*^6^,^7^,^9^. In addition, NBCn1 has four optional structural elements, namely cassettes I−IV, derived from alternative splicing of four of the five cassette exons. Finally, *Slc4a7* is able to express two types of products containing just the isolated Nt domain of NBCn1, caused by omitting the fifth cassette exon^9^.

*Slc4a10* encoding the electroneutral NBCn2 (*aka* NCBE) is known to contain two promoters, responsible for expressing two types of NBCn2 with different initial Nt ends starting with “MEIK” versus “MCDL^10^”. In addition, previous studies have shown that *Slc4a10* has two major cassette exons, namely inserts A and B^10^,^11^. Inclusion of insert A results in the expression of cassette A of 30 amino acids (aa) in the Nt domain of NBCn2. Alternative splicing of insert B causes the expression of four different carboxyl terminal ends (Ct): one long Ct (PDZ-Ct) with a typical PDZ-binding motif recognizable by PDZ-domain containing scaffold proteins, and three short Cts (non-PDZ-Ct) without PDZ-binding motif^10^,^11^. So far, 10 major NBCn2 variants (NBCn2-A through -J) have been identified^10^,^11^,^12^,^13^.

The diversity of the expression products of *Slc4* genes is of great physiological relevance. The structural variations could be related to the functional modulation of the transporters in the following aspects: (1) the developmental-specific expression; (2) spatial-specific (tissue- and cell-type-specific) expression; (3) establishing the intrinsic activity; (4) modulating the interaction with protein partners. For example, the optional cassette II of NBCn1 contains a binding site for Ca^2+^/calmodulin-activated serine/threonine phosphatase calcineurin Aβ^14^,^15^. A functional study shows that cassettes II, III, and IV of NBCn1 can stimulate the intrinsic activity and surface expression of the transporter in *Xenopus* oocytes^9^. The expression of particular NBCn2 variants is tissue specific. For example, in rodents, NBCn2 variants with the non-PDZ-Ct (e.g., NBCn2-A) are predominantly expressed in the CNS^10^,^13^ where NBCn2 is very abundantly expressed^11^,^12^,^16^,^17^,^18^. However, NBCn2 variants with the PDZ-Ct (e.g., NBCn2-C and -G) are predominantly expressed in epithelial tissues such as the kidney, choroid plexus in the brain, and reproductive tracts^10^.

In the previous study by Liu *et al.* mentioned above^10^, the expression of MCDL-NBCn2 was readily detected by western blotting from rat kidney. However, it was very difficult to amplify the cDNA encoding MCDL-NBCn2 from rat kidney by reverse transcription polymerase chain reaction (RT-PCR) with primers complimentary to the known 5′-UTR, suggesting that rat *Slc4a10* contain a third promoter. In the present study, we therefore performed 5′-rapid amplification of cDNA ends (5′-RACE) with total RNA from rat kidney and identified a novel promoter of *Slc4a10* expressing MCDL-NBCn2. Moreover, we found that this new promoter was able to express a group of new NBCn2 variants containing a large truncation in the Nt domain. We also identified a novel NBCn1 variant containing a large truncation in the Nt in addition to a novel full-length NBCn1 clone. In a heterologous expression system, we examined the consequences of the Nt truncation and isolated Nt domain on the membrane trafficking of the transporters.

## Results

### Expression of Slc4a10 in rat tissues—Identification of new promoter

**Figure 1:**
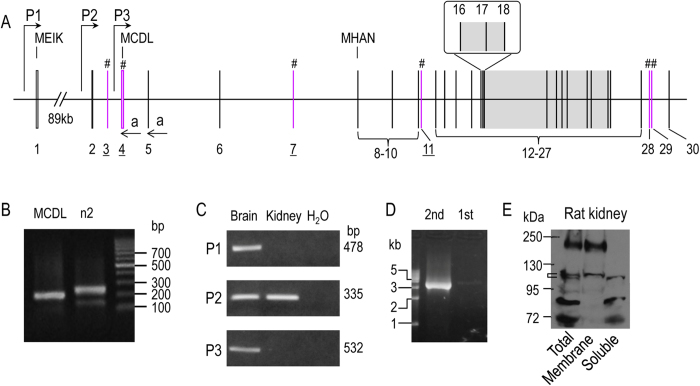
Expression of *Slc4a10* in rat. (**A**) Structure of rat *Slc4a10* gene. P1, P2, and P3 represent the three alternative promoters of rat *Slc4a10*. Promoter P2 and exon 3 are newly identified in the present study. The gray portion (exons 16–26) encodes the predicited transmembrane domain of NBCn2. # indicate the cassette exons (exons 3, 4, 7, 11, 27, 28) that can be alternatively spliced. (**B**) 5′-RACE to clone the 5′-UTR of NBCn2 transcripts from adult rat kidney. The reverse primer complimentary to exon 4 (arrow in panel **A**) was used to amplify the 5′-UTR of MCDL-NBCn2, and the reverse primer complimentary to exon 5 (arrow in panel A) was used to amplify the 5′-UTR of NBCn2. (**C**) RT-PCR analysis of the expression of NBCn2 transcripts in the brain and kidney of adult rat. Promoter-specific sense primers were used to amplify the NBCn2 transcripts derived from the three different promoters of *Slc4a10*. The anti-sense primer was the same for all three PCRs. The PCR products were verified by DNA sequencing. H_2_O was used as template for the control. (**D**) Cloning of full-length NBCn2 derived from promoter P2 by nested RT-PCR with rat kidney. (**E**) Western blotting of the distribution of NBCn2 proteins in cellular fractions of rat kidney homogenate.

In a previous study, we have shown that *Slc4a10* contains two promoters, enabling the expression of two types of NBCn2 with different extreme Nt starting with “MEIK” or “MCDL^10^”. By 5′-RACE and cDNA cloning with total RNA preparations from rat kidney, we identified two new exons (exons 2 and 3 in Fig. 1A) of *Slc4a10*. The new exon 2 of *Slc4a10* mapped from 46394706 to 46394776 in contigAC_000071.1, whereas the new exon 3 mapped from 46406628 to 46406642 in contig AC_000071.1. In addition to the two previously identified promoters (P1 and P3 in Fig. 1A), we found in the present study that rat *Slc4a10* contains a third promoter (P2, upstream of exon 2).

Figure 1B shows the result of a 5′-RACE experiment. Two sets of primers were used for the 5′-RACE: one specific for MCDL-NBCn2 (1st lane), and the other specific for both MEIK- and MCDL-NBCn2 (2nd lane). With the primers specific for MCDL-NBCn2, a product of ~200 bp was obtained. With the primers specific for both MEIK- and MCDL-NBCn2, a major product of ~250 bp plus a minor product of ~150 bp was obtained. The 5′-RACE products were subcloned into a vector and transformed into bacteria. Colonies were randomly selected for sequencing.

From the product in lane “MCDL”, we identified a *Slc4a10* transcript containing exons 2 + 4 encoding “MCDL-NBCn2”. From the product in lane “n2”, we identified three different types of *Slc4a10* transcripts: (1) a first one transcribed from promoter P1 containing exons 1 + 5 encoding MEIK-NBCn2; (2) a second one transcribed from promoter P2 containing exons 2 + 4 + 5 encoding MCDL-NBCn2; (3) a third one transcribed from promoter P2 containing exons 2 + 5, presumably encoding MHAN-NBCn2.

We performed RT-PCR analysis to examine the expression of the three alternative promoters of *Slc4a10* in the brain and kidney of adult rat. As shown in Fig. 1C, transcripts derived from promoters P1 and P3 were detectable in the brain, but not in the kidney. On the other hand, transcripts derived from promoter P2 were detectable in both the brain and the kidney. The results indicate that all three promoters are active in rat brain. However, it is promoter P2 that is predominantly expressed in the kidney.

We then performed nested RT-PCR with rat kidney using sense primers specific to exon 2 and anti-sense primers specific to the 3′-untranslated region (3′-UTR) of rat *Slc4a10* transcripts and obtained a product of ~3.5 kb (Fig. 1D). From this product, we identified several different *Slc4a10* transcripts, some encoding MCDL-NBCn2 (see “Novel Slc4a10 transcripts encoding MCDL-NBCn2”), the others encoding Nt-truncated NBCn2 variants starting with “MHAN” (see “Novel Slc4a10 transcripts encoding Nt-truncated MHAN-NBCn2”).

Finally, we examined the expression and cellular distribution of NBCn2 in rat kidney by western blotting with anti-NBCn2-Ct antibody directed against the unique Ct of rat NBCn2-C (Fig. 1E). The higher bands at ~180 kDa in “Total” and “Membrane” likely represented the glycosylated full-length NBCn2 proteins. The band at ~110 kDa in “Membrane” likely represented the matured Nt-truncated MHAN-NBCn2 (predicted molecular weight MW = 103.3 kDa), whereas the band at ~100 kDa in “Soluble” fraction likely represented the immature form of MHAN-NBCn2. The bands with MW lower than 90 kDa in the lanes “Total” and “Soluble” are likely the degradation products of NBCn2.

**Figure 2:**
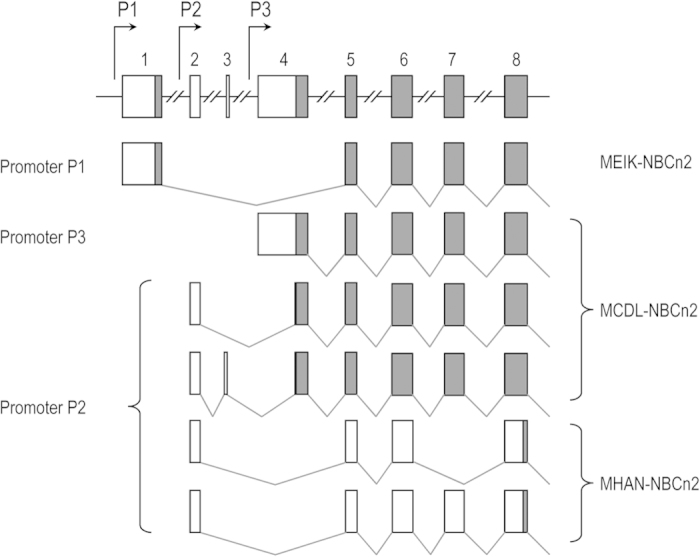
Exon structures (partial) of *Slc4a10* transcripts. Three types of transcripts could be expressed under the control of three different promoters of *Slc4a10*. The open boxes represent the untranslated regions, whereas the grayed boxes represent the coding region. The transcripts derived from promoter P1 are predicted to encode MEIK-NBCn2. Those derived from P3 are predicted to encode MCDL-NBCn2. Derived from promoter P2, the transcripts containing exon 4 are predicted to encode MCDL-NBCn2, whereas those lacking exons 4 or 4 + 7 are predicted to encode MHAN-NBCn2.

Figure 2 summarizes the exon structures of rat *Slc4a10* transcripts encoding three different types of Nts of NBCn2. MEIK-NBCn2 is encoded by the transcripts transcribed from promoter P1 of *Slc4a10*. MCDL-NBCn2 could be encoded by three types of transcripts, one derived from promoter P3 and two from P2. Finally, MHAN-NBCn2 could be encoded by two types of transcripts lacking either exon 4 or exons 4 + 7 derived from promoter P2.

### Alternative splicing of exon 28 of Slc4a10

In the present study, we found that the previously-identified exon 28 of *Slc4a10* was omitted in some transcripts of rat *Slc4a10* (see section “Novel Slc4a10 transcripts encoding Nt-truncated MHAN-NBCn2”). The finding represents the identification, for the first time, of a novel alternative splicing cassette in the Ct domain of NBCn2. We designated this novel cassetteas cassette C, being consistent with the designation of previously identified cassettes A (exon 11 in Fig. 1A) and B (exon 29 in Fig. 1A; for review, see ref. 1).

### Novel Slc4a10 transcripts encoding MCDL-NBCn2

In total, we obtained three novel full-length *Slc4a10* transcripts, all derived from promoter P2, that encode a MCDL-NBCn2 protein identical—regardless the optional Ala residue (Ala^256^)—to the previously identified NBCn2-G (accession #JX073717) which was derived from promoter P3. The three novel transcripts were:

(1) Accession #KJ452197 containing exon 3,the product of which contained Ala^256^.

(2) Accession #KM209338 containing exon 3, the protein product of which lacked Ala^256^.

(3) Accession #KF305251 lacking exon 3, the product of which contained Ala^256^.

### Novel Slc4a10 transcripts encoding Nt-truncated MHAN-NBCn2

By RT-PCR with rat kidney using primers specific to exon 2 of *Slc4a10*, we consistently obtained some full-length NBCn2 transcripts lacking exon 4 (the exon encoding the “MCDL” module). In addition, we obtained some partial clones lacking exons 4 + 7. These transcripts could be translated into a novel type of NBCn2 starting with “MHAN” using an alternative start codon in exon 8.

**Figure 3:**
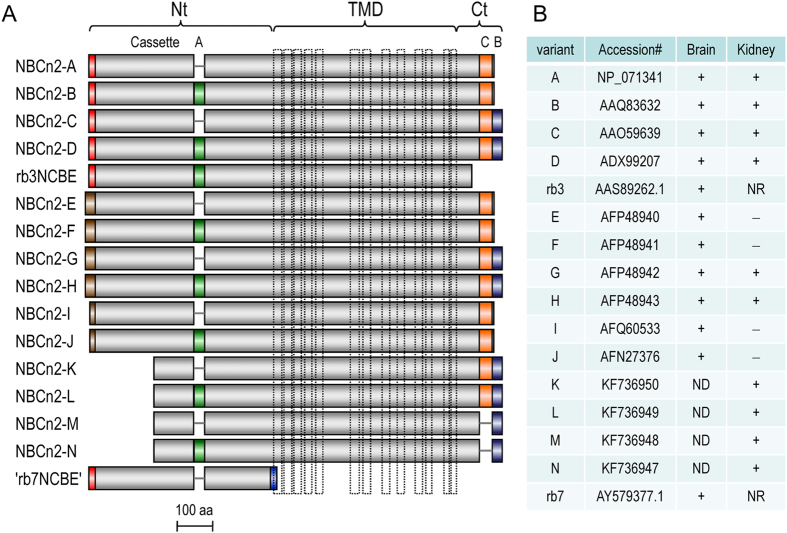
Structural diagram and expression of NBCn2 variants. (**A**) Diagram of primary structure of known NBCn2 variants. The vertical dashed-lines in TMD indicate the predicted transmembrane helices. (**B**) Summary of tissue distribution of NBCn2 variants according to previous reports^10^,^13^,^34^, the present study as well as database records in GenBank. NBCn2-A and -B were identified from human kidney^34^, but not from mouse kidney in the previous study^10^ or rat kidney in the present study. NR: not reported.

In total, we identified four such MHAN-NBCn2 variants: NBCn2-K (accession #KF736950), NBCn2-L (accession #KF736949), NBCn2-M (accession #KF736948), and NBCn2-N (accession #KF736947). Figure 3 summarizes the structural features of all known NBCn2 variants. As shown in Fig. 1E, western blotting analysis suggests that the Nt-truncated MHAN-NBCn2 is expressed at the protein level in rat kidney.

Note that, our cDNA cloning indicates that MHAN-NBCn2 represents a significant fraction of *Slc4a10* transcripts from rat kidney derived from promoter P2. For example, in one batch of cDNA screening, 8 over 29 clones were MHAN-NBCn2, whereas the rest 21 were MCDL-NBCn2. In another independent batch of screening for a PCR product obtained by using a different set of primers, the majority of the clones were MHAN-NBCn2. These observations indicate that MHAN-NBCn2 is relatively abundant in rat kidney.

### Alternative splicing of cassette B of Slc4a10 in kidney

In the present study, all variants identified from rat kidney, including MEIK-NBCn2 derived from promoter P1 (data not shown), MCDL-NBCn2 and MHAN-NBCn2 derived from promoter P2, lacked cassette B (exon 29). Thus, all NBCn2 variants identified from rat kidney would have the long PDZ-Ct. The results were consistent with the previous observation that, in the kidney of mouse and rat, cassette B is predominantly spliced out^10^.

### Characterization of transcriptional activities of promoters of rat Slc4a10

The expression of three types of NBCn2 transcripts with distinct 5′-UTR shows that *Slc4a10* contains three promoters. The previous study by Liu *et al.* has shown that NBCn2 variants with distinct Nt exhibit different tissue specificity in the central nervous system and the kidney^10^. Here, we examined, by using luciferase reporter assay, the cell specificity of transcriptional activities of the three promoters of rat *Slc4a10* in human embryonic kidney cell HEK293, rat ganglion cell RGC-5, and neuro-2A cells which is a mouse neuroblastoma cell line.

**Figure 4:**
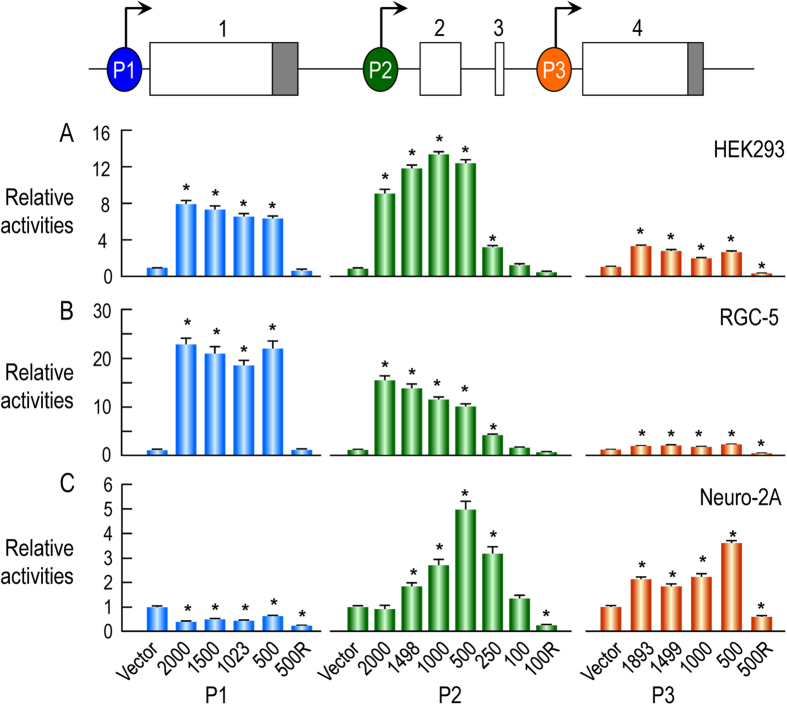
Luciferase reporter assay for relative transcription activities of promoters P1, P2, and P3 of rat *Slc4a10* in HEK293 cells. (**A**), RGC-5 cells (**B**) and neuro-2A cells (**C**). The gray boxes indicate the coding regions, whereas the open boxes represent the untranslated regions. The nucleotide numberings for P1 and P3 were relative to the start codon “ATG” of the following exon, whereas those for P2 were relative to the 3′-end of exon 2. For transcription activity assay, the genomic DNA fragment was subcloned into pGL3 basic vector expressing the firefly luciferase reporter. The construct with alphabet “R” in the name contained the reverse sequence of the corresponding region. Data were presented as mean ± SE. Each bar represented the mean of at least three independent experiments, each containing quadruplicates for an individual construct. Stars indicate that the bars are significantly different from pGL3 basic by one-way ANOVA followed by Dunnett’s comparison using Minitab 16 (Minitab Inc., State College, PA,USA).

As shown in Fig. 4, the three promoters of rat *Slc4a10* each had a unique profile of relative transcriptional activities in the three different cell lines. In HEK293, promoters P1 and P2 were much more active than P3. For example, the highest relative transcription activities of promoter P1 (bar “2000”, left panel of Fig. 4A) and P2 (bar “1000”, middle panel of Fig. 4A) were about 8 and 13 times higher than that of the control pGL3 basic vector, respectively, whereas the highest activity of P3 (bar “1893”, right panel of Fig. 4A) was 3.2 times higher than the control pGL3 basic. Similarly, promoters P1 and P2 elicited much higher transcription activities than P3 did in RGC-5 cells (Fig. 4B). In neuro-2A cells, promoter P1 had no detectable transcription activity inasmuch as all bars were significantly lower than the control vector (left panel in Fig. 4C). The highest transcription activities of P2 (bar“500”, middle panel in Fig. 4C) and P3 (bar“500”, right panel in Fig. 4C) were about 5 and 3.6 times of that of control vector, respectively.

### Novel NBCn1 variants with new alternative Nt ends

**Figure 5:**
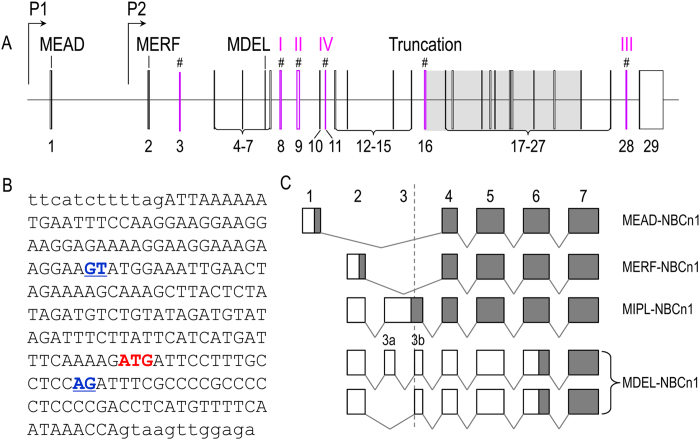
Identification of novel mouse *Slc4a7* transcripts encoding MIPL-NBCn1 and MDEL-NBCn1. (**A**) Structure of mouse *Slc4a7* gene. *Slc4a7* contains two alternative promoters as demonstrated previously^9^. In addition, the gene contains 29 exons, of which exon 3 was newly identified in the present study. # indicates cassette exons (exon 3,8,9, 11,16,28). (**B**) Nucleotide sequence of exon 3 (upper case) and the flanking intron (lower case) of mouse *Slc4a7*. The underlined (blue) indicate the cryptic splicing donor and acceptor. The “ATG” in red indicates the alternative start codon for the “MIPL” motif. (**C**) Diagram to show the exon structure of *Slc4a7* transcripts encoding the three different types of Nt ends of NBCn1. The grayed boxes represent the coding regions, whereas the open boxes represent the non-coding regions. The originally-described transcripts, predicted to encode MEAD-NBCn1, are derived from promoter P1. The originally-described MERF- as well as the newly identified MIPL- and MDEL-NBCn1 are derived from promoter P2. In the present study, exon 3 was identified only in the transcripts derived from promoter P2 of *Slc4a7*.

*Slc4a7* encoding the electroneutral Na^+^/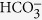 cotransporter NBCn1 contains two promoters and is capable of expressing two types of NBCn1 variants starting with “MERF” or “MEAD^9^”. In the present study, by RT-PCR with mouse heart, testis, and uterus, we identified a new exon of *Slc4a7*, namely, exon 3 in Fig. 5A. This new exon contains a cryptic “intron” (Fig. 5B). Consider the cryptic “intron”, we designate the two remaining portions as exons 3a and 3b (Fig. 5C).

In our cDNA cloning, this new exon 3 was present just in some specific transcripts derived from promoter P2 of *Slc4a7*, and not in those derived from P1. As shown in Fig. 5C, four different splicing manners were identified for exon 3 in the P2-derived transcripts of *Slc4a7* from our cDNA cloning with mouse tissues: (1) splicing-out the entire exon 3; (2) splicing-in the entire exon 3; (3) splicing-in exons 3a + 3b; (4) splicing-in exon 3b only. Splicing-out the entire exon 3 would cause the expression of MERF-NBCn1 using the start codon in exon 2. The transcript containing the entire exon 3 would express a novel NBCn1 starting with “MIPL” using an alternative start codon located in the cryptic “intron” of exon 3. Inclusion of exons 3a + 3b or 3b only would cause the expression of MDEL-NBCn1 using an alternative start codon in exon 6.

In the present study, one NBCn1 variant starting with “MIPL” (NBCn1-R, accession# KF279521) was identified from mouse heart. In addition, one NBCn1 variant starting with “MDEL” (NBCn1-Q, accession# KF279520) was identified from mouse testis and uterus.

### Functional expressionof novel NBCn1 variants in oocytes

The full-length NBCn1-R and the Nt-truncated NBCn1-Q tagged with EGFP at the Ct end were expressed in *Xenopus* oocytes. An oocyte was perfused with nominally 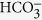-free ND96 solution, then exposed to 5% CO_2_/33 mM 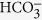 solution for ~10 minutes (min), then exposed to “0Na”solution with Na^+^ replaced with NMDG for ~5 min. The resting *V*_m_ of oocytes expressing NBCn1-R (−24.5 ± 1.1, p = 2.0 × 10^−6^ vs H_2_O) and NBCn1-Q (−24.2 ± 1.2, p = 1.5 × 10^−5^ vs H_2_O) was significantly more depolarized than that of H_2_O-injected oocytes (−47.5 ± 3.0). Furthermore, the resting pH_i_ of oocytes expressing NBCn1-R (7.26 ± 0.03, p = 0.015 vs H_2_O) and NBCn1-Q (7.31 ± 0.03, p = 0.024 vs H_2_O) was also significantly different from that of H_2_O-injected oocytes (7.17 ± 0.05).

**Figure 6:**
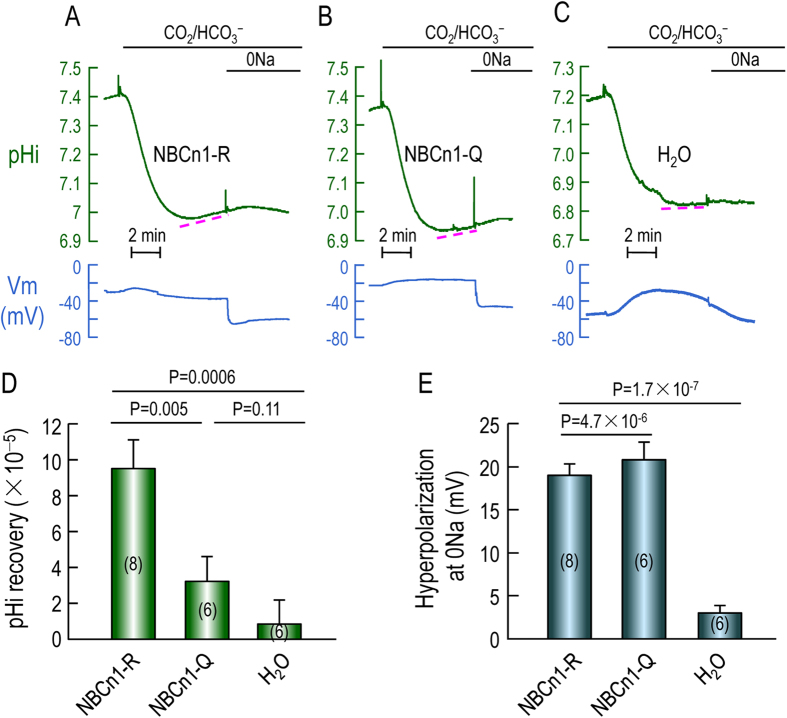
Functional expression of novel NBCn1 variants in *Xenopus* oocytes. (**A**) Representative recordings of pH_i_ and *V*_*m*_ from an oocyte expressing NBCn1-R. (**B**) Representative recordings of pH_i_ and *V*_*m*_ from an oocyte expressing NBCn1-Q. (**C**) Representative recordings of pH_i_ and *V*_*m*_ from a H_2_O-injected oocyte. (**D**) Summary of pH_i_ recovery rate (dpHi/dt) upon CO_2_-induced intracellular acidification in oocytes. (**E**) Summary of hyperpolarization upon removal of extracellular Na^+^. NBCn1-R and -Q were tagged with EGFP at the Ct. pH_i_ recovery rate represents the slope of pH_i_ trace like those indicated by the dashed lines in panels **A**–**C**. Numbers in parentheses indicate the number of oocytes represented for each bar. One-tailed unpaired students’ T-test was performed for statistical analysis.

As shown in Fig. 6A, the oocytes expressing full-length NBCn1-R elicited significant Na^+^-dependent pH_i_ recovery upon CO_2_-induced intracellular acidification compared to H_2_O-injected control oocytes. The pH_i_ recovery rate of the oocytes expressing NBCn1-Q, which was significantly lower than that of oocytes expressing NBCn1-R, tended on average to be greater than that of H_2_O-injected cells although did not reach statistical significance. Removal of extracellular Na^+^ induced similar extents of hyperpolarization for NBCn1-R and NBCn1-Q (Fig. 6B).

As demonstrated previously, the depolarization in resting *V*_m_ of *Xenopus* oocytes and the hyperpolarization induced by removal of extracellular Na^+^ are characteristic of NBCn1^7^. Our results suggest that both NBCn1-R and NBCn1-Q exhibit characteristic NBCn1 action but that the functional expression of NBCn1-Q is significantly less than that of NBCn1-R. Biotinylation of one of the batches of cells from which these data were gathered (data not shown), reveals that differences in the plasma membrane expression of NBCn1-Q vs NBCn1-R are likely to underlie the lower functional expression of Q vs R.

### Effects of Nt-truncation on cellular localization of NCBTs in neuro-2A cells

**Figure 7:**
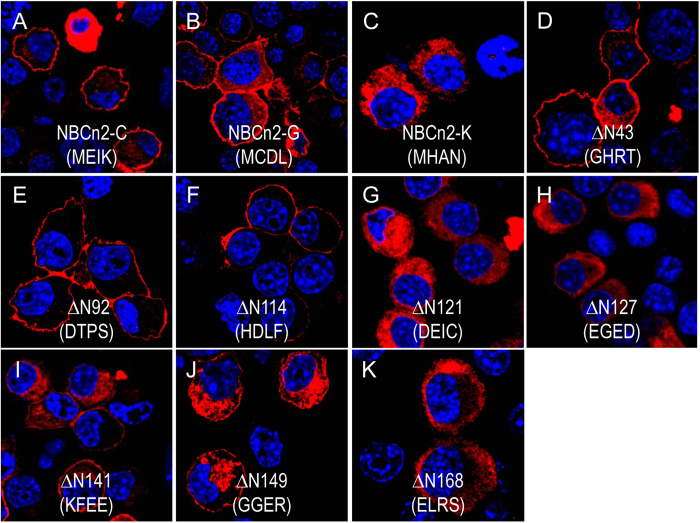
Cellular localization of NBCn2 variants and mutants heterologously expressed in neuro-2A cells. (**A**) NBCn2-C (starting with MEIK). (**B**) NBCn2-G (starting with MCDL). (C) NBCn2-K (starting with MHAN). (**D**–**K**) NBCn2 mutants truncated to various positions in the Nt domain. As an example, ΔN43 (truncated to “GHRT”) represents a mutant with the first 43 aa residues removed based on NBCn2-C. The cDNA encoding NBCn2 variants or mutants was subcloned into pcDNA3.1. The cells were transfected with the corresponding construct, incubated for 24 hours for transient expression, and then fixed with 4% PFA for immunocytochemistry. NBCn2 proteins were probed with anti-NBCn2-Ct antibody in combination with Dylight 549 goat-anti-rabbit secondary antibody.

We then examined the effect of truncation in the Nt domain on the membrane trafficking of different NCBTs in neuro-2A cells. When heterologously expressed in neuro-2A cells, rat NBCn2-C and -G were both well localized in the plasma membrane (Fig. 7A,B). However, rat NBCn2-K, the natural Nt-truncated variant, was nearly all retained in the cytosol (Fig. 7C), although its surface localization was occasionally observed in very few cells (data not shown). The same was true for the natural Nt-truncated variant rat NBCn2-M when expressed in neuro-2A cells (data not shown). The results suggested that the Nt portion omitted in MHAN-NBCn2 contain information critical for efficient surface expression of the transporter. To test this hypothesis, we made a series of truncation mutations to the Nt of NBCn2-C and tested the cellular localization of these constructs in neuro-2A cells.

As shown in Fig. 7D–F (constructs ΔN42 starting with “MGHRT”, ΔN92, ΔN114), removal of the first 114 residues based upon NBCn2-C had no significant effect on the surface expression of the transporter proteins in neuro-2A cells. However, both ΔN121 (“DEIC”, Fig. 7G) and ΔN127 (“EGED”, Fig. 7H) were virtually all retained in the cytosol of neuro-2A cells. The results suggest that the sequence “HDLFTEL” could play an important role in the surface localization of rat NBCn2 in neuro-2A cells. Surprisingly, starting from mutant ΔN127, further deletion (mutants ΔN141 and ΔN149 in Fig. 7I–J) largely restored the surface expression of the transporter. Based upon ΔN149, deleting 19 more residues (ΔN168 or “ELRS”, Fig. 7K) again resulted in cytosol retention, a phenotype similar to that of NBCn2-K.

**Figure 8:**
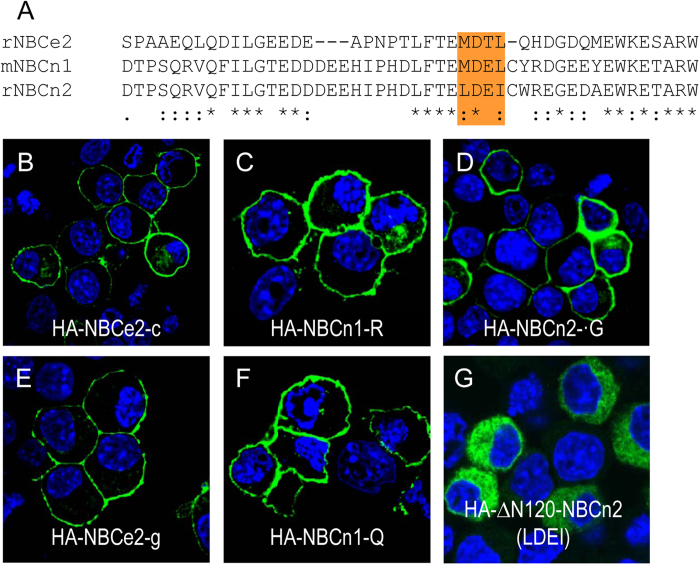
Cellular localization of NBCe2, NBCn1, and NBCn2 heterologously expressed in neuro-2A cells. (**A**) Sequence alignment of the Nt of rat NBCe2, mouse NBCn1, and rat NBCn2. (**B**–**D**) Cellular localization of the full-length rat HA-NBCe2-c (**B**), mouse HA-NBCn1-R (**C**), and rat HA-NBCn2-G (D). (**E**−**G**) Cellular localization of the Nt-truncated rat HA-NBCe2-g (E), mouse HA-NBCn1-Q (**F**), and mutant rat HA-ΔN120-NBCn2 truncated to “LDEI” (**G**). HA (“YPYDVPDYA”) was tagged at the Nt end of the NCBT proteins. Mouse anti-HA primary antibody in combination of Alexa 488 goat-anti-mouse secondary antibody was used to detect the localization of HA-tagged proteins.

Taken together, the above observations indicate that the initial Nt portion has a complicated effect on the surface expression of NBCn2. *Slc4a5* is shown to be able to produce an Nt-truncated variant, i.e., NBCe2-g (*aka* NBC4g) starting with “MDTL”^19^. This NBCe2-g is homologous to the newly identified NBCn1-Q starting with “MDEL” as well as NBCn2 mutant ΔN121 starting with “MDEI” in terms of the truncation in the Nt (Fig. 8A). To compare the effects of Nt-truncation on the surface expression of NBCe2, NBCn1, and NBCn2, we generated variants of rat NBCe2, mouse NBCn1, and rat NBCn2 with hemagglutinin (HA) tagged at the Nt.

When heterologously expressed in neuro-2A cells, the full-length variants HA-NBCe2-c, HA-NBCn1-R, and HA-NBCn2-G were all well localized in the plasma membrane (Fig. 8B–D). In addition, both Nt-truncated HA-NBCe2-g and HA-NBCn1-Q were well expressed in the plasma membrane, suggesting that the Nt-truncations had no significant effect on the cellular localization of these two variants (Fig. 8E,F). In contrast, HA-ΔN120-NBCn2 was all retained in the cytosol (Fig. 8G), consistent with the observation with ΔN121 in Fig. 7G.

Taken together, the results showed that the homologous truncation in the Nt domain elicited distinct effect on the cellular localization among NBCe2, NBCn1, and NBCn2.

### Effects of coexpressing full-length NBCn2 on the cellular localization of Nt-truncated NBCn2

**Figure 9:**
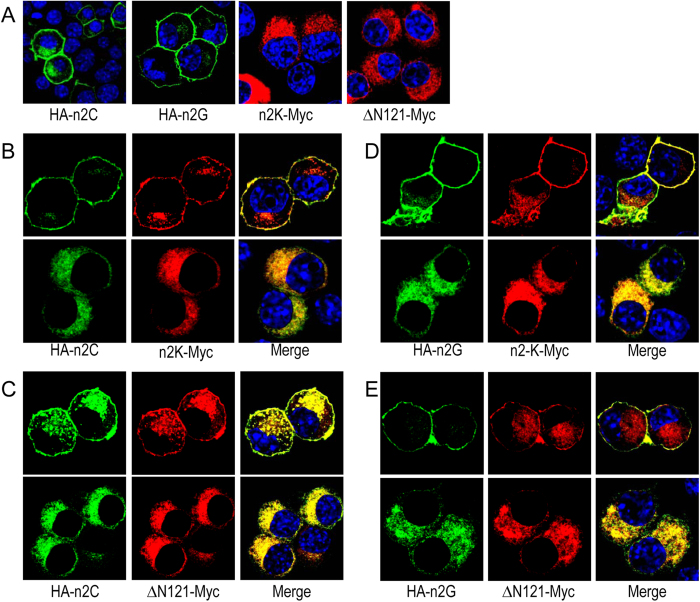
Effect of coexpressing full-length NBCn2 on the cellular localization of Nt-truncated NBCn2 in neuro-2A cells. (**A**) Full-length and Nt-truncated NBCn2 individually expressed in neuro-2A cells. (**B**−**E**) Coexpression of HA-NBCn2-C and NBCn2-K-Myc (**B**), HA-NBCn2-C and ΔN121-Myc (C), HA-NBCn2-G and NBCn2-K-Myc (D), HA-NBCn2-G and ΔN121-Myc (**E**) in neuro-2A cells. Full-length NBCn2-C and -G were HA-tagged at the Nt end. Nt-truncated NBCn2-K and mutant “ΔN121” were tagged with Myc (EQKLISEEDL) at the Ct end. Full-length NBCn2 was probed with anti-HA, whereas the Nt-truncated NBCn2 was probed with anti-Myc. Two populations of cells were observed for each case in panels **B**−**E**. In a minor population of cells, both full-length and Nt-truncated NBCn2 were primarily colocalized on the plasma membrane. In the other major population of cells, both full-length and Nt-truncated NBCn2 were primarily colocalized in the cytosol.

To examine the effect of coexpressing the full-length NBCn2 on the cellular localization of the Nt-truncated NBCn2, we generated constructs encoding rat NBCn2-C or -G tagged with HA at the Nt ends as well as constructs encoding rat NBCn2-K or mutant “ΔN121” tagged with Myc at the Ct ends. When heterologously expressed individually in neuro-2A cells, both HA-NBCn2-C and HA-NBCn2-G were primarily distributed in the plasma membrane (two panels left to Fig. 9A), whereas NBCn2-K-Myc and “ΔN121-Myc” were virtually all retained in the cytosol (two panels right to Fig. 9A). These observations were consistent with the results from the corresponding constructs in Fig. 7.

When coexpressed in neuro-2A cells, HA-NBCn2-C and NBCn2-K-Myc were always colocalized but with distinct cellular distribution patterns in two different populations of cells. In a minor population of cells (estimated to account for ~8% of the total cells positive in NBCn2 staining), HA-NBCn2-C and NBCn2-K-Myc were colocalized in the plasma membrane (three upper panels in Fig. 9B). In the vast majority of cells (estimated to account for ~92% of the total cells positive in NBCn2 staining), HA-NBCn2-C and NBCn2-K-Myc were colocalized in the cytosol (three lower panels in Fig. 9B). The colocalization suggests that HA-NBCn2-C and NBCn2-K-Myc likely form heterodimers when simultaneously expressed in neuro-2A cells.

Similarly, when coexpressed in neuro-2A cells, HA-NBCn2-C and the Nt-truncated NBCn2 mutant “ΔN121-Myc” were colocalized in the plasma membrane of a minor population of cells (upper panels in Fig. 9C), but were both retained in the cytosol in the vast majority of cells positive in NBCn2 expression (lower panels in Fig. 9C). Similar results were obtained for HA-NBCn2-G coexpressed with NBCn2-K-Myc (Fig. 9D) or “ΔN121-Myc” (Fig. 9E), as well as for wild-typeNBCn2-C or -G (with no tag) coexpressed withNBCn2-K-Myc orΔN121-Myc (data not shown).

**Figure 10:**
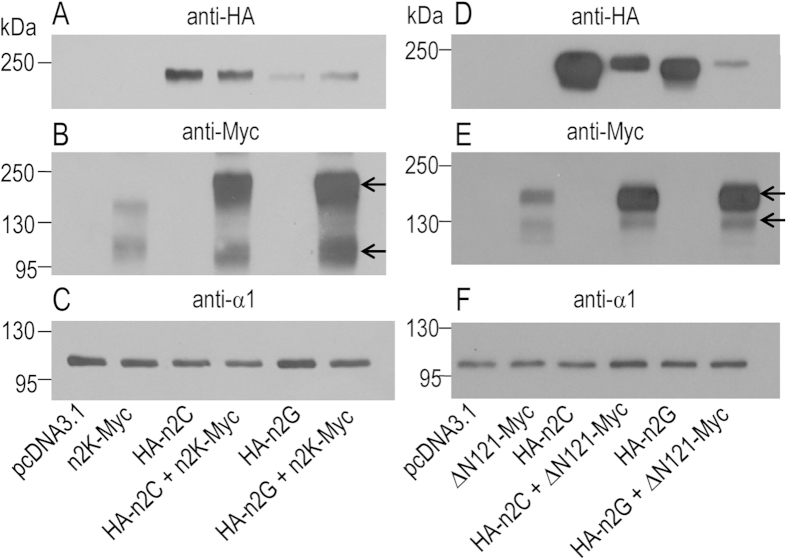
Biotinylation analysis of the effect of coexpressing the full-length NBCn2 on the surface abundance of Nt-truncated NBCn2 in neuro-2A cells. **(A,D)** Expression of full-length NBCn2 (n2C and n2G) with HA tagged at the Nt end. (**B,E**) Expression of Nt-truncated NBCn2 (n2K and ΔN121) with Myc tagged at the Ct end. (**C,F**) Expression of endogenous Na^+^-K^+^-ATPase probed with anti-α1 to verify equal loading in each lane. Surface proteins were prepared by biotinylation and separated on 10% SDS-PAGE for western blotting.

The above observations suggest that the presence of the full-length NBCn2 enhance the surface localization of the Nt-truncated NBCn2-K and ΔN121 at least in a specific population of cells. We performed biotinylation assay with neuro-2A cells to further examine this effect of full-length NBCn2. Figure 10A shows that, when coexpressed with the Nt-truncated NBCn2-K-Myc, the relative surface abundance of the full-length HA-NBCn2-C and -G was not substantially changed. However, the relative surface abundance of NBCn2-K-Myc was greatly increased in the presence of HA-NBCn2-C or -G (Fig. 10B). The staining of Na^+^-K^+^ ATPase showed the equal loading for each lane (Fig. 10C).

Similarly, the presence of the full-length HA-NBCn2-C or HA-NBCn2-G substantially enhanced the surface abundance of the mutant ΔN121-MyC in neuro-2A cells (Fig. 10D–F). Note that, in the presence of ΔN121-Myc, the surface abundance of HA-NBCn2-C or HA-NBCn2-G was greatly decreased compared to the case in the absence of ΔN121-Myc. This last observation was consistent with the fact that, when coexpressed with the mutant ΔN121-Myc, the full-length NBCn2 proteins were retained in a major population of cells (Fig. 9).

**Figure 11:**
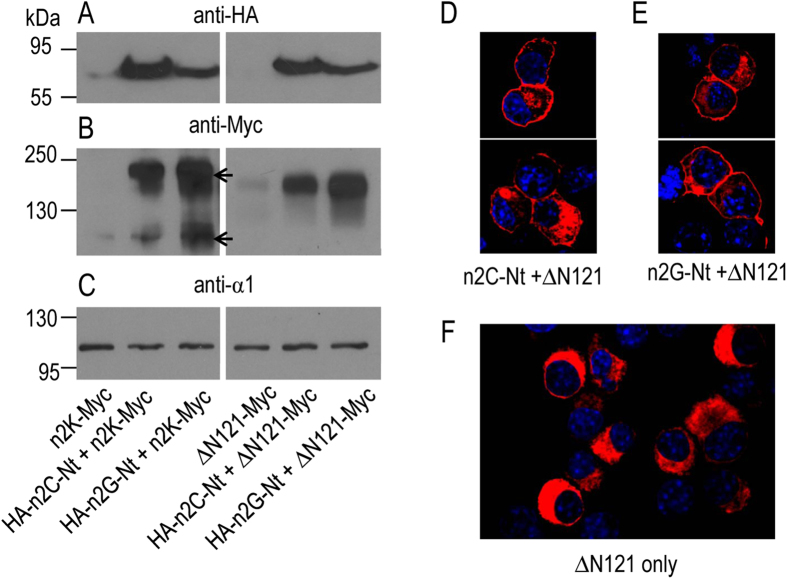
Effect of isolated Nt domain on surface expression of Nt-truncated NBCn2 in neuro-2A cells. (**A**) Expression of isolated Nt domain of rat NBCn2-C/G. Whole cell lysate was used for the analysis of the expression of the isolated Nt domains. (**B**) Biotinylation analysis of the surface expression of Nt-truncated NBCn2. (**C**) Biotinylation analysis of the surface expression of Na^+^-K^+^-ATPase. (**D–E**) Cellular localization of mutant ΔN121in neuro-2A cells in the presence of the isolated Nt domain of NBCn2-C or NBCn2-G. (**F**) Cellular localization of mutant ΔN121 in the absence of the isolated Nt domain of NBCn2 in neuro-2A cells. The isolated n2C-Nt consisted of the sequence of “MEIK…FLSQ” of rat NBCn2-C, and n2G-Nt consisted of the sequence of “MCDL…FLSQ” of rat NBCn2-G. For western blotting analysis, n2C-Nt and n2G-Nt were tagged with HA at the Nt end, whereas the Nt-truncated NBCn2-K and ΔN121 were tagged with Myc at the Ct end. In immunocytochemistry analysis, the isolated Nt and Nt-truncated NBCn2 proteins had no tag. The cells were stained with anti-NBCn2-Ct polyclonal antibody.

Finally, we examined the effect of expressing the isolated Nt domain of NBCn2-C or -G on the surface abundance of the Nt-truncated NBCn2 proteins. Strikingly, the presence of just the Nt domain of NBCn2-C or -G was sufficient to substantially increase the surface abundance of the Nt-truncated NBCn2-K-Myc andΔN121-Myc (Fig. 11A–C). Consistently, immunocytochemistry staining showed that, in the presence of the isolated Nt domain, the Nt-truncated ΔN121 was well expressed in the plasma membrane of a specific population of cells (Fig. 11D–E), different from that without coexpression of the isolated Nt domain (Fig. 11F).

Note that, two bands were detected in each lane expressing the Nt-truncated NBCn2-K-Myc or mutant ΔN121-Myc (Figs 10 & 11). The two bands presumably represented the fully glycosylated (higher band) and core-glycosylated (lower band) NBCn2 proteins, respectively^17^. Interestingly, the MW of the presumed “fully glycosylated” form of NBCn2-K-Myc coexpressed with full-length HA-NBCn2-C or -G was slightly higher than that of the presumed glycosylated form of NBCn2-K-Myc when expressed alone (Fig. 10B). The results suggest that, in the presence of full-length NBCn2, the Nt-truncated NBCn2-K obtain more complicated modification, presumably higher degree of glycosylation. It is also noteworthy that the MW of the lower bands of NBCn2-K-Myc (Figs 10B & 11B) was very close to that of the presumed “MHAN-NBCn2” from rat kidney in Fig. 1E.

Taken together, our data suggest that, when simultaneously expressed in neuro-2A cells, the full-length HA-NBCn2-C or -G preferably form heterodimers with the Nt-truncated NBCn2-K-Myc or mutant ΔN121-Myc. This heterodimerization could enhance the surface expression of the Nt-truncated NBCn2-K or mutant “ΔN121”. Moreover, the isolated Nt domain of the full-length NBCn2 was sufficient to enhance the surface expression of the Nt-truncated NBCn2 proteins.

### Bimolecular fluorescence complementation assay for interaction between NBCn2 variants

Bimolecular fluorescence complementation (BiFC) is a strategy initially developed to determine the interaction between two proteins^20^. The nonfluorescent fragments of the Nt and Ct halves of enhanced yellow fluorescent protein (YFP) are linked to two different proteins. The two complementary fragments of Nt and Ct are able to form fluorescent YFP if they are tethered by protein interaction^20^,^21^.

**Figure 12:**
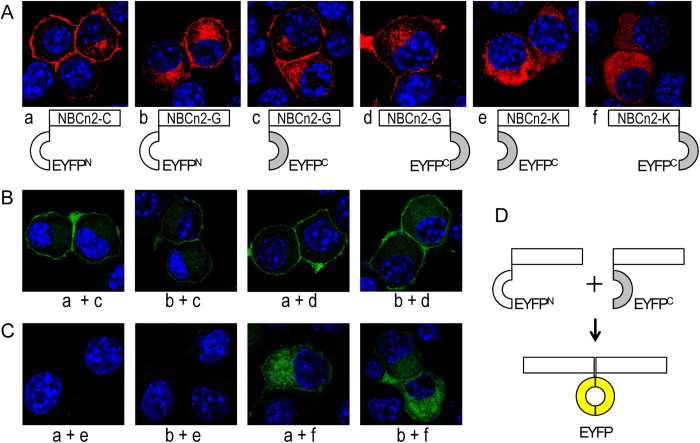
Bimolecular fluorescence complementation (BiFC) assay. The amino-terminal fragment (1–158 aa, YFP^N^) or carboxyl-terminal fragment (159–238 aa, YFP^C^) of YFP were fused to NBCn2 variants at their Nt (e.g., YFP^N^-NBCn2-C) or Ct end (e.g., NBCn2-G-YFP^C^). The fusion proteins were transiently expressed in neuro-2A cells. (**A**) Individual expression of NBCn2 variants fused with YFP^N^ or YFP^C^. NBCn2 were probed with anti-NBCn2-Ct in combination of Dylight 549 goat-anti-rabbit secondary antibody and visualized by laser confocal microscopy. (**B**) Paired coexpression of full-length NBCn2-C and -G fused with YFP^N^ or YFP^C^. (**C**) Paired coexpression of full-length NBCn2-C or G fused with YFP^N^ and NBCn2-K fused with YFP^C^. (**D**) Diagram to show the principal of BiFC assay. In panels **B** and **C**, the cells were fixed with 4% PFA and stained with DAPI. YFP was visualized directly by laser confocal microscopy. In panel **B**, strong fluorescence signal was seen for both Nt-Nt pairs (YFP^N^ and YFP^C^ were both tagged at the Nt of NBCn2, e.g., YFP^N^-NBCn2-C plus NBCn2-G-YFP^C^ in “a + c”) and Nt-Ct pairs (YFP^N^ and YFP^C^ were tagged at the Nt and Ct of NBCn2, respectively, e.g., YFP^N^-NBCn2-G plus NBCn2-G-YFP^C^ in “b + d”). However, in panel **C**, fluorescence signals were seen only for Nt-Ct pairs, but not for Nt-Nt ones.

Here we employed BiFC to investigate the potential interaction between NBCn2 variants. A series of constructs were generated encoding NBCn2 variants tagged at either the Nt or Ct end with the complementary nonfluorescent amino-terminal fragment (1–158 aa, YFP^N^) or the carboxyl-terminal fragment (159–238 aa, YFP^C^) of YFP. Fig. 12A shows the tagging of YFP fragments did not significantly change the cellular distribution of NBCn2 variants in neuro-2A cells compared to the corresponding ones shown above. For example, the full-length NBCn2-C and -G fused with complementary YFP fragments were primarily localized in the plasma membrane (four panels left to Fig. 12A), whereas NBCn2-K with YFP^C^ linked to its Nt or Ct was nearly all retained in the cytosol (two panels right to Fig. 12A).

Different variants were paired for coexpression in neuro-2A cells to investigate the formation of NBCn2 heterodimers. Figure 12B shows the pairs between two full-length NBCn2 variants. Strong fluorescent signals were observed for the Nt-Nt pairs, i.e., YFP^N^ and YFP^C^ were both linked to the Nt of NBCn2 variants, such as the cases in “a + c” and “b + c”. The results suggest that the two NBCn2 molecules form dimer via interaction between their Nt domains. In addition, fluorescent signals were observed for the Nt-Ct pairs, i.e., YFP^N^ and YFP^C^ were linked to the Nt and Ct of two NBCn2 molecules, respectively, as were in “a + d” and “b + d”. The results suggest that the Nt and Ct domains of NBCn2 are in close proximity, enabling the formation of fluorescent YFP from two complementary fragments.

For the pairs between the full-length and the Nt-truncated NBCn2 variants (Fig. 12C), fluorescent signals were observed for the Nt-Ct pairs (Nt of NBCn2-C/G and Ct of NBCn2-K in “a + f” and “b+f”), but not the Nt-Nt pairs (“a + e” and “b + e”). The presence of fluorescent signals from the Nt-Ct pairing is consistent with the Nt-Ct pairing between full-length NBCn2 variants in Fig. 12B. In the Nt-Nt pairing between NBCn2-C (or -G) with NBCn2-K, the two complementary fragments of YFP could spatially be not close enough due to the great mismatch in the length of the Nts of the two types of NBCn2 variants—the Nt of NBCn2-K was 182 (or 194) aa shorter than that of NBCn2-C (or -G). Therefore, the two complementary fragments of YFP could not form fluorescent protein even the two Nt domains of the full-length and Nt-truncated NBCn2 variants could interact with each other.

### Coimmunoprecipation of full-length NBCn2 or isolated Nt with Nt-truncated NBCn2

**Figure 13:**
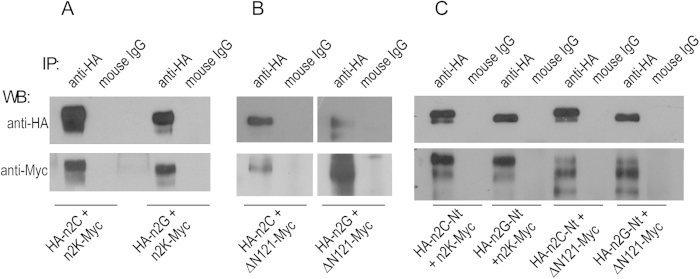
Co-immunoprecipitation of Nt-truncated NBCn2 with full-length NBCn2 or isolated Nt domain. Myc-tagged Nt-truncated NBCn2-K or mutant ΔN121 was coexpressed with HA-tagged full-length variants or the isolated Nt domain in Neuro-2A cells. Cell lysate was prepared as described in Methods and immunoprecipitated with anti-HA antibody. (**A**) Co-immunoprecipitation of NBCn2-K-Myc and HA-NBCn2-C or -G. (**B**) Co-immunoprecipitation of ΔN121-Myc and HA-NBCn2-C or -G. (**C**) Co-immunoprecipitation of NBCn2-K-Myc and ΔN121-Myc with isolated Nt of NBCn2-C or -G.

We performed immunoprecipation, using the mouse anti-HA antibody, with the lysate of neuro-2A cells simultaneously expressing the full-length NBCn2 or isolated Nt with the Nt-truncated NBCn2. As shown in Fig. 13A&B, NBCn2-K-Myc and ΔN121-Myc was coimmunoprecipitated with HA-NBCn2-C or -G. Similar results were obtained with immunoprecipitation using rabbit anti-Myc antibody (data not shown). Again, these observations were consistent with the idea that the Nt-truncated NBCn2-K or mutant ΔN121 forms heterodimers with the full-length NBCn2-C or -G when coexpressed in neuro-2A cells. Finally, we could immunopreciptate NBCn2-K-Myc and ΔN121-Myc with the isolated Nt domain of NBCn2-C or -G tagged with HA (Fig. 13C), suggesting protein-protein interaction between the isolated Nt domain and the Nt-truncated NBCn2.

## Discussion

The present study expands our knowledge about the transcriptome of the electroneutral Na^+^/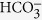 cotransporters NBCn1 and NBCn2. In summary, *Slc4a7*encoding NBCn1 contains two promoters: the distal promoter P1 located upstream of exon 1 and the proximal P2 located in the intron following exon 1^9^. In addition, on the transcript level, *Slc4a7* contains six cassette exons that can be alternatively spliced, namely, exons 3 (newly identified from mouse in the present study), 8,9,11,16, and 28.

The new exon 3, present in transcripts derived from promoter P2 of *Slc4a7*, contains a cryptic intron and an alternative initiator codon. The new exon 3 could be spliced in four different ways: (1) splicing-out the entire exon; (2) splicing-in the entire exon; (3) splicing-in exon 3a + 3b; (4) splicing-in exon 3b only.

Summarized below are the major structural variations identified so far in NBCn1 variants:

• Four alternative Nt ends: MEAD vs MERF vs MIPL vs MDEL. MEAD-NBCn1 is derived from promoter P1, whereas the other three are derived from promoter P2. “MIPL” and “MDEL” represent the two new alternative Nts identified in the present study. MIPL-NBCn1 is translated from the *Slc4a7* transcripts containing the entire exon 3, whereas MDEL-NBCn1 is translated from the *Slc4a7* transcripts containing exon 3a + 3b or 3b only. In addition to the four major alternative Nt ends, one more minor variation in the Nt of NBCn1 is an extension of four residues (“VTSR”) in the MEAD module identified from human tissues^9^. This minor variation is probably species specific.
• Four major alternative cassettes: cassettes I (11 aa, encoded by the 3′-portion of exon 8), II (123 aa in rodent and 124 aa in human, encoded by exon 9), III (36 aa, encoded by exon 28), IV (20 aa, encoded by exon 11)^7^,^9^.

So far, 18 NBCn1 variants have been identified: NBCn1-A through -R, among which NBCn1-Q and -R are newly identified in the present study. These 18 variants all contain the three major domains of the Nt (regardless of truncation such as NBCn1-Q), TMD, and Ct. In addition to these TMD-containing variants, one more major structural variation of NBCn1 is the expression of some *Slc4a7* products containing just the isolated Nt domain caused by splicing-out exon 16^9^.

To summarize, *Slc4a10* encoding NBCn2 contains three promoters: P1, P2, and P3, among which P2 is newly identified in the present study. In addition, on the transcript level, *Slc4a10* contains six major cassette exons that can be alternatively spliced in or out, including exons 3, 4, 7, 11, 28 and 29. On the protein level, the major structural variations in NBCn2 variants include:

- Three alternative Nts starting with “MEIK, “MCDL” (in rat), or “MHAN”. MEIK-NBCn2 is derived from promoter P1. MCDL-NBCn2 can be expressed from either P2 or P3. Finally, MHAN-NBCn2 is expressed from promoter P2. MHAN-NBCn2 is a truncation version of MCDL-NBCn2 due to splicing-out exon 4 or exons 4 + 7.
- Cassette A in the Nt domain. The cassette A is a 30-aa structural element encoded by exon 11 that is homologous in position to the 20-aa cassette IV of NBCn1.
- Cassette C in the Ct domain newly identified in the present study. This 39-aa cassette encoded by exon 28 of *Slc4a10* is homologous in sequence to the cassette III of NBCn1.
- Variations in the extreme Ct arising from alternative splicing of cassette B (exon 29). Exclusion of the entire cassette B results in the expression of the long Ct containing a PDZ-binding motif, whereas inclusion of different portions of cassette B results in the expression of several different short Cts without PDZ-binding motif^10^. Note that, an isolated variant rb3NCBE with a truncated Ct was reported in GenBank. This clone lacks exons 27–29.

Finally, NBCn2 contains one more minor variation, i.e., the optional inclusion of a single Ala residue in the Nt domain due to usage of a cryptic splicing donor site for exon 10. The presence/absence of this single Ala residue appears to be random and presumably does not have a major effect on the function of NBCn2.

Consider the major structural variations in the protein products, at least 15 NBCn2 variants, NBCn2-A through -N plus “rb3NCBE”, have been reported. These 15 variants all contain the three major domains of the Nt (regardless of truncation such as NBCn2-K), TMD, and Ct (regardless of the truncation such as “rb3NCBE”). In addition to these TMD-containing NBCn2 variants, a variant “rb7NCBE” containing just the isolated Nt domain of NBCn2 was reported in GenBank.

**Figure 14:**
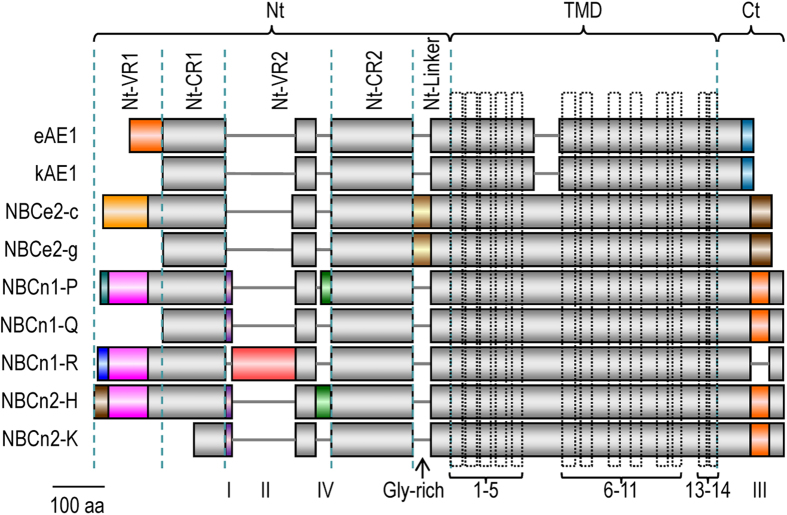
Structural comparison of variants of selected Slc4 members. The diagram was drawn based upon a sequence alignment for rat eAE1 (accession #NP_036783.2), rat kAE1 (accession #AAH85748.1), rat NBCe2-c (accession #NP_997677.1), rat NBCe2-g (accession #BAM73282.1), mouse NBCn1-P (accession #AFI43932.1), mouse NBCn1-Q (accession #AGX13876.1), mouse NBCn1-R (accession #AGX13877.1), rat NBCn2-H (accession #JX073718), rat NBCn2-K (accession #AHG54969.1). The sequence alignment was performed with online multiple sequence alignment tool Clustal Omega from European Bioinformatics Institute of EMBL (http://www.ebi.ac.uk/Tools/msa/clustalo/). The Nt domains of the Slc4 transporters consist of two variable regions VR1 and VR2, as well as two conserved regions CR1 and CR2. Cassettes I through IV indicate the four optional structural elements of NBCn1. Compared to eAE1, kAE1 is truncated by 78 aa at the Nt. Compared to NBCe2-c, NBCe2-g is truncated by 115 aa. NBCn1-Q is truncated by 125 aa compared to NBCn1-P. NBCn2-K is truncated by 194 aa compared to NBCn2-H.

Among the novel variants of NBCn1 and NBCn2 identified in the present study are a group of special ones that contain large truncations in the Nt domain. The Nt domain of the Slc4 transporters contains two conserved regions Nt-CR1 and Nt-CR2 as well as two variable regions Nt-VR1 and Nt-VR2^22^. As shown in Fig. 14, NBCn1-Q lacks the whole Nt-VR1. Two more such Slc4 variants lacking Nt-VR1 have been reported: kAE1 identified from the kidney^23^, and NBCe2-g identified from the choroid plexus in the brain^19^. Compared to NBCn1-Q, kAE1, and NBCe2-g, MHAN-NBCn2 is truncated by ~60 more residues in the Nt, causing the loss of about half of Nt-CR1.

In the present study, when heterologously expressed in *Xenopus* oocytes, NBCn1-Q lacking Nt-VR1 clearly exhibits the electrical signs that are characteristic NBCn1 activity. A paucity of plasma membrane expression made it impractical to demonstrate significant Na^+^/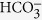 cotransport activity using our standard assay, but we note that the resting pH_i_ of oocytes expressing NBCn1-Q was more alkaline than that of H_2_O-injected cells which would be consistent with a low level of HCO_3_^−^ transport activity. An alternative approach will be required to conclusively demonstrate Na^+^/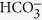 cotransport activity by NBCn1-Q. Moreover, when heterologously expressed in neuro-2A cells, in addition to the full-length variants NBCe2-c, NBCn1-P, NBCn2-C and NBCn2-G, the Nt-truncated NBCn1-Q and NBCe2-g are both well localized on the plasma membrane. The membrane localization of NBCe2-g in neuro-2A cells is consistent with a previous demonstration that NBCe2-g is active when heterologously expressed in HEK293^19^. kAE1, the kidney AE1 analogous to NBCe2-g and NBCn1-Q in terms of the truncation in the Nt domain, is also active when heterologously expressed in *Xenopus* oocytes^24^,^25^. Taken together, the above observations from literature as well as the present study show that, Nt-VR1 in the Nt domain is not essential for the function (normal surface expression and transport activity) of AE1, NBCe2, and NBCn1.

A preliminary study shows that removing residues 1–92 does not impair the functional expression of human NBCn2-B in *Xenopus* oocytes^26^. Consistent with this observation, in the present study, removal of the first 43 or 92 residues (constructs ΔN43 and ΔN92 in Fig. 7) has no significant effect on the surface expression of rat NBCn2 in neuro-2A cells. Moreover, we extend the observation by showing that removal of the first 114 residues (ΔN114) has no significant effect on the surface expression of rat NBCn2 in neuro-2A cells. Taken together, we conclude that the region1–114 is not essential for the surface expression, and presumably the activity of NBCn2.

However, mutant ΔN121 (starting with “DEIC”), which is truncated by just 7 more residues (“HDLFTEL”) based upon ΔN114, is primarily retained in the cytosol of neuro-2A cells. The results suggest that the sequence “HDLFTEL” contain information critical for the surface localization of NBCn2 in neuro-2A cells. Note that, ΔN121 is an artificial NBCn2 version analogous to kAE1, NBCe2-g, and NBCn1-Q in terms of the Nt truncation. It is surprising that similar truncation in the Nt has distinct effects on the surface expression of theseSLC4 transporters.

It is intriguing that removing 20 more residues (“DEICWREGEDAEWRETARWL”) based upon mutant ΔN121 partially restores the surface expression (construct ΔN141). Further truncation, e.g., ΔN168 and the natural variant NBCn2-K, causes severe cellular retention in neuro-2A cells. Interestingly, an Nt-truncated mutant of NBCe1-C with the first 213 aa removed is able to be delivered to the plasma membrane, although it is inactive when heterologously expressed in *Xenopus* oocytes^27^. This truncation in NBCe1-C, which is 39 aa more downstream than the truncation in NBCn2-K, causes the loss of Nt-VR1 plus almost the entire Nt-CR1 of NBCe1.

The present study provides evidence for the formation of heterodimers between NBCn2 variants. An increasing amount of evidence shows that the Slc4 family transporters function as dimers in the plasma membrane. Firstly, crystallographical studies show that the cytosolic Nt domains of AE1 and NBCe1 are dimers^28^,^29^,^30^. Secondly, using multiple biochemical approaches, Kao *et al.* have shown that NBCe1-A endogenously expressed in mouse kidney as well as heterologously expressed in HEK293 cells predominantly form dimers^31^. Thirdly, using BiFC approach, Chang *et al.* are able to observe strong fluorescence when YFP^N^ (1–158) and YFP^C^ (159–238), the nonfluorescent complementary fragments of YFP, are tagged to the Nt ends of differnet two molecules of NBCe1-A expressed in *Xenopus* oocytes, suggesting the formation of homodimers between NBCe1-A individuals^21^. Finally, by using spatial fluorescence intensity fluctuation analysis, Sergeev *et al.* have shown evidence that NBCe1-A forms dimer *in vivo*^32^.

Here, we make several lines of observations consistent with the notion that NBCn2 proteins form homodimers (between two molecules of the same NBCn2 variant) or heterodimer (between two different NBCn2 variants).

Firstly, immunocytochemistry staining shows that, when simutaneously expressed in neuro-2A cells, the full-length NBCn2-C or -G preferably colocalizes with the Nt-truncated NBCn2-K or mutant ΔN121, either in plasma membrane or in cytosol. The colocaliozation suggests that the full-length NBCn2 and the Nt-truncated NBCn2-K or ΔN121 interact with each other, presumably forming heterodimers.

Secondly, our coimmunoprecipation study shows that the full-length NBCn2-C (or NBCn2-G) interacts with the Nt-truncated NBCn2-K or ΔN121, a fact consistent with the formation of heterodimers between the full-length NBCn2 with the Nt-truncated NBCn2.

Thirdly, our BiFC assay provides evidence that NBCn2 proteins form homo- or heterodimers in neuro-2A cells. The fluorescence observed from the full-length NBCn2 pairs tagged at the Nt with complimentary YFP fragments (such as YFP^N^-NBCn2-C + YFP^C^-NBCn2-G or YFP^N^-NBCn2-G + YFP^C^-NBCn2-G) suggests that the two NBCn2 molecules form dimer via interaction between the two Nt domains. Moreover, the Nt domain of one NBCn2 molecule is likely in close proximity with the Ct domain of the other inasmuch as fluorescence is visible from the pairs of NBCn2 with YFP^N^ tagged at the Nt (such as YFP^N^-NBCn2-C or YFP^N^-NBCn2-G) and that with YFP^C^ tagged at the Ct (such as NBCn2-G-YFP^C^ or NBCn2-K-YFP^C^). Note that, Chang *et al.* claims that only minimum fluorescence is visible when YFP^N^ and YFP^C^ are linked to the Nt and Ct ends of NBCe1-A, respectively^21^.

Note that, when simultaneously expressed in neuro-2A cells, the full-length NBCn2 and the Nt-truncated ones (NBCn2-K or mutant ΔN121) are always colocalized either in the plasma membrane or in the cytosol. Therefore, it appears that, when coexpressed, the full-length NBCn2 and the Nt-truncated ones (such as NBCn2-K or mutant ΔN121) preferably form heterodimers. Interestingly, NBCn2-K coexpressed with the full-length NBCn2 variants has a higher MW compared to NBCn2-K expressed individually in neuro-2A cells. The presence of the full-length NBCn2 presumably benefits the maturation, e.g., by promoting the glycosylation of the truncated NBCn2, therefore enhancing its surface expression.

The heterodimerization between the full-length NBCn2 and the Nt-truncated ones could be physiologically relevant. If the Nt-truncated NBCn2 variants newly identified from rat kidney are functional as a transporter, we would expect that they are expressed in the plasma membrane *in vivo*. Unusually, these natural variants are extremely poorly expressed on the plasma membrane in neuro-2A cells. The presumable heterodimerization with the full-length NBCn2 could significantly enhance the surface expression of the Nt-truncated ones (such as NBCn2-K) in a specific population of cells. The heterodimerization that is beneficial to the surface localization of Nt-truncated NBCn2 could explain the detection of MHAN-NBCn2 proteins in the membrane fraction of rat kidney.

It is noteworthy that, some unusual products containing just the isolated Nt domain have been reported for specific NCBT members, such as NBCn1^9^, NBCn2, and NDCBE (for review, see ref. 1). The observations that the isolated Nt domain of NBCn2 interacts with and is sufficient to enhance the surface abundance of the Nt-truncated NBCn2 suggest that the isolated Nt domain of NCBT could act as a regulatory unit to modulate the function of the transporter.

In conclusion, we make the following novel findings in the present study.

(1) We identify several novel variants of NBCn1 and NBCn2, including a group of variants (MDEL-NBCn1 and MHAN-NBCn2) containing a large truncation in the Nt domain. Moreover, we find a novel alternative cassette, i.e., cassette C in the Ct of NBCn2, which is homologous to cassette III of NBCn1. These findings expand our knowledge about transcriptome of *Slc4a7* and *Slc4a10*.

(2) The Nt-truncated MHAN-NBCn2as well as the mutant ΔN121 is primarily retained in the cytosol when heterologously expressed by their own in neuro-2A cells. However, the surface abundance of MHAN-NBCn2 and mutant ΔN121 is greatly enhanced in the presence of the full-length NBCn2, likely by forming heterodimers with the full-length variants.

(3) We show for the first time that the isolated Nt domain interacts with and promotes the surface localization of the Nt-truncated NBCn2 proteins.

Presently, it is not clear whether the Nt-truncated MHAN-NBCn2 is functionally active in 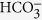 transport. It appears to be practically difficult to functionally characterize the MHAN-NBCn2 due to it’s the poor surface expression when expressed alone. Even in the presence of full-length NBCn2 or the isolated Nt-domain, the Nt-truncated MHAN-NBCn2 or mutant ΔN121 is visible in the plasma membrane of only a very small fraction of the total cells that are positive for NBCn2 expression.

We should also note that, our observations about membrane trafficking of the transporters were made in heterologous expression systems. Different cell types might have their own mechanisms governing the maturation and trafficking of membrane proteins. The native cells in mammalian tissues might have their specific mechanism to assist more efficiently the membrane trafficking of MHAN-NBCn2 that was poorly expressed in the plasma membrane of our heterologous system. Nevertheless, our observations about protein interaction and its effect on the membrane trafficking of the transporters are likely applicable to some extent in native mammalian tissues, and are therefore likely to have physiological relevance.

## Experimental Procedures

### Cloning of 5′-UTR of rat Slc4a10 transcripts

5′-rapid amplification of cDNA ends (5′-RACE) was performed with 5′-Full RACE Kit (cat# D315, TaKaRa Biotechnology Co., Ltd., Dalian, China) to amplify the 5′-untranslated region (5′-UTR) of the cDNAs encoding rat NBCn2. Briefly, total RNA was isolated from the kidney of adult Sprague Dawley rats with TRIzol® Reagent (cat#15596-018, Life Technologies Corporation, Carlsbad, CA, USA) according to the manufacturer’s instructions. Following decapping and ligation with an adaptor provided with the kit, the RNA was used for cDNA synthesis with anti-sense primer 5′-GTGATGATGCTG-3′complimentary to rat *Slc4a10*. Nested polymerase chain reaction (PCR) was then performed to amplify the 5′-UTR of rat *Slc4a10* transcripts. The first round of PCR was performed with sense primer 5′-CATGGCTACATGCTGACAGCCTACTG-3′ and anti-sense primer 5′-GTGCTCCTCATCATCGTCCTCAGTTC-3′. The second round of PCR was performed with sense primer 5′-actact*cccggg*ACAGCCTACTGATGATCAGTCGATG-3′ and anti-sense primer 5′-actact*gcggccgc*TGTTCCACCTCTATCCACAACGG-3′ (lower case representing the artificially-introduced sequence, italicized representing the restrictive sites for subcloning into vector). An anti-sense primer 5′-actact*gcggccgc*GACCTAGAGACTGGAAATGCTCACAG-3′was used instead in the second round of PCR to specifically amplify the 5′-UTR of *Slc4a10* transcripts derived from promoter P2. The PCR products were restricted, ligated with a vector, and then transformed into Top10 competent cells for screening.

### RT-PCR analysis of three promoters of rat Slc4a10

Reverse transcription PCR (RT-PCR) was performed with PrimeSTAR^TM^ HS DNA polymerase (cat#DR010S, TaKaRa Biotechnology Co., Ltd.) to examine the expression of NBCn2 transcripts derived from different promoters of *Slc4a10* in rat brain and kidney. Sense primer 5′-TGGTGAGTTGGAGTGTGCAGTTGCC-3′ was used for *Slc4a10* transcripts derived from promoter P1. Sense primer 5′-CTCCTCACATACAGTATTCAGGGCACAG-3′ was used for transcripts derived from promoter 2. The sense primer 5′-GGATGATGCACAGTGCTTGGGATACG-3′ was used for transcripts derived from promoter 3. The same antisense primer 5′-GTGCTCCTCATCATCGTCCTCAGTTC-3′ was used for the three different sets of PCR.

### Cloning of cDNA encoding rat NBCn2

The full length cDNA encoding rat MEIK-NBCn2 derived from promoter P1 was amplified by nested PCR with sense primer 5′-TGGTGAGTTGGAGTGTGCAGTTGCC-3′ plus anti-sense primer 5′-GGTGTTGACCTGCTCAGAGGCTGAAC-3′ for the first round of PCR, and sense primer 5′-atcg*cccggg*CCTGATCCGAATACTAAGCAGAGCG-3′ (lower case representing the non-*Slc4a10* sequence, italicized representing the restrictive site for the following subcloning) plus anti-sense primer 5′-atgact*gcggccgc*GGATGGGAGACAGGGCTTACAATGAC-3′ for the second round of PCR.

The full length cDNA encoding rat MCDL-NBCn2 derived from promoter P2 was amplified by nested PCR. The sense primers for the first and second rounds of PCR were 5′-CTCCTCACATACAGTATTCAGGGCACAG-3′ and 5′-gcat*cccggg*TTCAGGGCACAGAAATCTTTTGATTGAC-3′, respectively. The anti-sense primers were the same as those used for the cloning of rat MEIK-NBCn2.

The full length cDNA encoding rat MCDL-NBCn2 derived from promoter 3 was amplified by nested PCR. The sense primers for the first and second rounds of PCR were 5′-GGATGATGCACAGTGCTTGGGATACG-3′ and 5′-atgcat*cccggg*CTGTAGATGCTGAGAGACAGAGACG-3′, respectively. The anti-sense primers were the same as those used for the cloning of rat MEIK-NBCn2.

The PCR products were subcloned into a vector and then transformed into TOP10 cells for NBCn2 variant identification.

### Cloning of cDNA encoding mouse NBCn1

Nested PCR was performed with total RNA from C57BL/6J mouse tissues to amplify the NBCn1 cDNA derived from the promoter P2 of *Slc4a7*. Sense primer 5′-CACTGCCAGAAACAAGACCTACCCTG-3′ plus anti-sense primer 5′-ACAGTTACATGAAGAAAGCCCACAGAGAAGCC-3′ was used for the first round of PCR. Sense primer 5′-actact*cccggg*GCCAGAAACAAGACCTACCCTGTCAGTATTAC-3′ plus anti-sense primer 5′-actact*gcggccgC*ACCACATGGGCAGACTCCTTATTCTACC-3′ was used for the second round of PCR. The PCR products were restricted, subcloned into a vector, and transformed into Top 10 competent cells for the identification of NBCn1 variants.

### Luciferase reporter assay

Transcription activities were analyzed by luciferase reporter assay with Dual-Luciferase^®^ Reporter Assay Systems (cat#E1910, Promega Corporation, Madison, WI, USA) as described previously^10^. Briefly, the promoter regions of rat *Slc4a10* were amplified by PCR from rat genomic DNA and subcloned into pGL3 basic vector expressing firefly luciferase. Neuro-2A, human embryonic kidney cells (HEK293), and rat ganglion cells (RGC-5) were cultured in DMEM medium (Cat#11995, Life Technologies Corporation) supplemented with 10% fetal bovine serum (Cat#10099-133, Gibco®, Life Technologies Corporation) and 1% Penicillin/Streptomycin (Cat#15140-122, Life Technologies Corporation). Cells were transfected by Lipofectamine^TM^ 2000 (cat#11668, Life Technologies Corporation) with a mixture of the construct (derived from pGL3 basic) expressing firefly luciferase under the control of *Slc4a10* promoter and the construct pGL4.74 expressing renilla luciferase. The molar ratio of the two constructs was 50/1 (pGL3 over pGL4.74). The cells were then cultured for 24 hours (hrs), rinsed with PBS, and lysed with lysis buffer. 20 μL of LARII plus 20 μL of Stop&Glo^®^ Reagent was mixed with 10 μL of lysate and subjected immediately to fluorescence measurements on a GLOMAX^®^ 20/20 Luminometer (cat#E5311, Promega). The fluorescence intensity of firefly of each construct was first normalized to the intensity of the corresponding renilla. This firefly-to-renilla ratio of each construct was then normalized to that of pGL3 basic vector. The resulted value was used as an index for the transcription activity of the promoter.

### Antibodies

Rabbit polyclonal antibody anti-NBCn2-Ct directed against the PDZ-Ct of NBCn2 was generated by GenScript (Nanjing, China) with a synthetic peptide “IESRKEKKADSGKGVDRETC”. A cysteine was introduced at the Ct end for conjugation to keyhole limpet hemocyanin. The antibody was affinity-purified with the immunogen. Anti-α1 (directed against the α1 subunit of Na^+^-K^+^-ATPase) was purchased from Cell Signaling Technology (cat#3010, Danvers, MA, USA). Rabbit anti-Myc (cat#AE009), mouse anti-HA (cat#AE008) were purchased from Abclonal (Cambridge, MA, USA). Normal rabbit IgG (cat#sc-2027) and normal mouse IgG (cat#sc-2025) were purchased from Santa Cruz Biotechnology (Santa Cruz, CA, USA). Goat-anti-rabbit secondary antibody conjugated with horse radish peroxidase (HRP) was purchased from Thermo Scientific (Rockford, IL, USA). Goat-anti-mouse secondary antibody conjugated with HRP was purchased from Beyotime (cat#A0216, Beyotime, Haimen, China). Alexa 488 goat-anti-mouse (cat#705-545-003) from Jackson ImmunoResearch (West Grove, PA, USA) and Dylight 549 goat-anti-rabbit (cat#E032320-01) secondary antibodies were purchased from EarthOx (Millbrae, CA, USA).

### Construction of expression vector for Xenopus oocytes, preparation and injection of cRNAs

The expression construct of NBCn1 tagged with EGFP at Ct for *Xenopus* oocyte was generated starting from pGH19-rNBCn2-C-EGFP described previously^10^. The pGH19-rNBCn2-C-EGFP was digested with XmaI and AgeI. The vector fragment containing the cDNA encoding EGFP was purified by agarose gel electrophoresis. The cDNA encoding NBCn1 was amplified by PCR, double digested with XmaI and AgeI, and ligated with the pGH19 vector fragment to generate the expression construct. The resultant fusion protein contained a linker “CSPVAT” between NBCn1 and EGFP.

cRNA was prepared with T7 mMessage mMachine^®^ kit (cat#AM1344, Life Technologies Corporation) according to the manufacturer’s instruction. Oocytes of stages V–VI were injected with 25 ng cRNA and then incubated at 18 ^o^C in OR3 medium for 4–5 days prior to electrophysiology measurement.

### Electrophysiology measurement

Electrophysiology measurements for membrane potential (*Vm*) and intracellular pH (pH_i_) of oocytes were performed as reviewed previously^33^. Briefly, an oocyte was placed in a perfusion chamber and impaled with a proton-selective microelectrode filled with H^+^-ionophore I cocktail (Cat#95293, Sigma) and a *V*_*m*_-sensitive microelectrode filled with 3 M KCl. A third electrode filled with 3M KCl was placed in the bath close to the oocyte as reference. The signal of the electrodes was recorded using an FD223 dual-channel electrometer (World Precision Instruments, Inc., Sarasota, FL, USA) and an OC-275 oocyte clamp (Warner Instrument Corp., Hamden, CT, USA). Data were sampled every 500 ms.

Solutions used for electrophysiology recordings:

Nominally “HCO_3_^−^-free”ND96: (in mM) 96 NaCl, 2 KCl, 1 MgCl_2_, 1.8 CaCl_2_, and 5 HEPES, pH 7.50, 200 mOsm).

5% CO_2_/33 mM 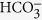: (in mM) 63NaCl, 2 KCl, 1 MgCl_2_, 1.8 CaCl_2_, and 5 HEPES. After adjusted to pH 7.5, the solution was added with 33 mM NaHCO_3_ and then bubbled with 5% CO_2_.

Na-free 5% CO_2_/33 mM 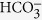 (0Na solution): (in mM) 66 N-methyl-d-glucamine (NMDG), 2 KCl, 1 MgCl_2_, 1.8 CaCl_2_, and 5 HEPES. After adjusted to pH 7.5, the solution was added with 33 mM NMDG and then bubbled with 5% CO_2_.

Data were acquired using custom software written by Dale Huffman from the laboratory of Walter Boron (Case Western Reserve University, Cleveland, OH, USA) and analyzed using Microsoft Excel.

### Membrane protein preparation from rat kidney and western blotting

Rat kidney tissue of ~300 mg was homogenized in 1 ml protein isolation buffer (in mM: 7.5 NaH_2_PO4, 250 sucrose, 5 EDTA, 5 EGTA, pH 7.0) containing 1% protease inhibitor cocktail (cat#P8340, Sigma-Aldrich, St. Louis, MO, USA) with a DY-89 II homogenizer (SCIENTZ, NingBo, China). The homogenate was centrifuged at 4,000 *g* for 10 min at 4 ^o^C to remove the cell debris. 20 μl of the supernatant was saved and used as “Total” protein. 800 μl supernatant was then ultracentrifuged at 100,000 *g* at 4 ^o^C for 1 hr. The resultant supernatant was saved as cytosol “Soluble” fraction. The pellet (“Membrane” fraction) was dissolved in 800 μl of protein resuspension buffer containing 20 mM Tris, 5 mM EDTA, 5% SDS, pH 8.0.

Equal volume of “Total”, “Soluble”, and “Membrane” proteins were separated on sodium dodecyl sulfate polyacrylamide gels (SDS-PAGE) and then transferred to a PVDF membrane. The membrane was blocked with 5% milk in 1×TBST (1 mM Tris, 150 mM NaCl, 0.1% Tween20, pH 7.4) for 1 hr at room temperature (RT). The membrane was then probed with primary antibody in 1% milk at 4 ^o^C overnight. The membrane was washed 5 times with 1×TBST, incubated with HRP-conjugated secondary antibody at a dilution of 1:5000 for 3 hrs at RT, and then washed 5 times with 1×TBST.Chemiluminescencewas performed with SuperSignal^®^ West Pico (Thermo Scientific) prior to X-ray exposure.

### Immunocytochemistry

The cDNAs encoding NBCe2, NBCn1, or NBCn2 were amplified by PCR, subcloned into pcDNA3.1(−), and transfected into neuro-2A cells with Lipofectamine^TM^ 2000 (Life Technologies Corporation). The cells were incubated for 24 hrs for transient expression, washed twice with PBS, and then fixed with 4% paraformaldehyde in PBS for 30 min at RT. Following 3 times wash with PBS, the cells were permeablized with TENT (50`mM Tris, 5 mM EDTA, 150 mM NaCl, 1% Triton X-100, pH 7.5) for 15 min at RT, and blocked with 15% normal goat serum in 28 mM PBS containing 450 mM NaCl, 0.3% Triton X-100, pH7.4, for 30 min at RT. The cells were incubated with primary antibody (at a dilution of 1:100) at 4 ^o^C overnight, washed with PBS for three times, and then incubated with secondary antibody at a dilution of 1:100 for 1 hr at RT. The cells were then washed three times with PBS, and counter-stained with 4,6-diamidino-2-phenylindole dihydrochloride (DAPI). Images were acquired with Fluoview FV1000 confocal laser scanning microscope (Olympus, Tokyo, Japan).

### Biotinylation

Membrane protein was prepared using Pierce® Cell Surface Protein Isolation Kit (cat#89881, Thermo Scientific) according to the manufacture’s instruction. Briefly, the cells were washed twice with BupH^TM^ PBS (100 mM sodium phosphate, 150 mM NaCl; pH 7.2) and then incubated with EZ-Link® Sulfo-NHS-SS-Biotin for 30 min at 4 ^o^C. The cells were rinsed with quenching solution (100 mM sodium phosphate, 50 mM glycine, pH 7.4) for five times. The cells were then collected and lysed with 500 ul lysis buffer (100 mM sodium phosphate, 150 mM NaCl, pH 7.2, 1% Triton-X-100) containing 1% protease inhibitor cocktail for 30 min on ice. The lysate was centrifuged at 10,000 × *g* for 2 min at 4 ^o^C.100 μl NeutrAvidin® Agarose was then added to the supernatant, incubated for 1 hr at RT, and then washed with lysis buffer for 4 times. The biotin-labeled protein bound to NeutrAvidin® Agarose was eluted with 100 ul sample buffer (62.5 mM Tris-HCl, pH 6.8, 1% SDS, 10% glycerol, 50 mM DTT) for 1 hr at RT and used for western blotting analysis.

#### Co-immunoprecipitation

Neuro-2A cells were cotransfected with pcDNA3.1(−) containing the cDNA encoding Nt-truncated NBCn2 tagged with Myc at the Ct end and that containing the cDNA encoding full-length NBCn2 or just the isolated Nt domain tagged with hemagglutinin (HA) at the Nt end. The cells were then incubated at 37 ^o^C for 24 hr for transient expression, collected and lysed with 1 ml IP buffer (Cat#P0013, Beyotime, Haimen, China) for 30 min on ice. The lysate was centrifuged at 12,000 *g* for 10 min at 4 ^o^C. The supernatant was divided into two aliquots, one added with 2 μl anti-HA or anti-Myc antibodies, the other added with 2 μl normal mouse as control, then incubated at 4 ^o^Covernight. Each aliquot was then added with 50 μl Protein A/G PLUS beads (cat#2003, Santa Cruz Biotechnology, Santa Cruz, CA, USA) and incubated for another 2 hr at 4 ^o^C. The beads were collected by centrifugation at 1000 *g* for 2 min and rinsed with 1ml IP buffer for 6 times. The beads were added with 40 μl 1× SDS sample buffer, boiled at 98 ^o^C for 5 min, and centrifuged at 10,000 *g* for 1 min. The supernatant was saved for western blotting analysis.

### Bimolecular fluorescence complementation assay

Bimolecular fluorescence complementation (BiFC) assay was performed as described previously^21^, with slight modification. Briefly, the cDNA encoding the YFP Nt (1–158 aa, YFP^N^) or Ct (159–238 aa, YFP^C^) fragments was amplified from pmCitrine-C1 containing cDNA encoding a variant of YFP, and was fused in frame to the cDNA encoding rat NBCn2 variants. The resultant fragment was subcloned into pcDNA3.1(−). When the YFP fragments (YFP^N^ or YFP^C^) were added to the Nt end of NBCn2, a linker “GTEEAL” was introduced between YFP fragment and NBCn2. When YFP^C^ was added to the Ct end of NBCn2, a linker “CSPVAT” was introduced between NBCn2 and YFP^C^. The constructs containing the cDNA encoding the fusion proteins were transfected into neuro-2A cells for transient expression. The cells were fixed with 4% PFA in PBS, counter-stained with DAPI. The cells were then visualized on an Olympus FV1000 microscope with excitation = 488 nm, and emission = 500/100 nm.

## Additional Information

**How to cite this article**: Wang, D.-K. *et al.* Effects of Nt-truncation and coexpression of isolated Nt domains on the membrane trafficking of electroneutral Na^+^/HCO_3_^–^ cotransporters. *Sci. Rep.* **5**, 12241; doi: 10.1038/srep12241 (2015).
